# Systematic Review on the Impact of Various Types of Universal Basic Income on Mental Health in Low- and Middle-Income Countries

**DOI:** 10.3390/bs14080726

**Published:** 2024-08-20

**Authors:** Simona Gomboc, Matija Zagoranski, Anaja Kos, Tinkara Bolta, Teodora Kitanovska, Gaja Rupena, Lara Slabanja, Julija Soklič, Lara Ružič Povirk, Lina Šenica, Elara Udvanc, Tija Vrhovnik, Matej Kržišnik, Vesna Jug

**Affiliations:** Faculty of Mathematics, Natural Sciences and Information Technologies, University of Primorska, Titov Trg 4, SI-6000 Koper, Slovenia; simona.gomboc@upr.si (S.G.);

**Keywords:** universal basic income, unconditional cash transfers, mental health, low-income countries, middle-income countries

## Abstract

In the past two decades, a significant surge in interest in investigating mental disorders and challenges faced by low- and middle-income countries in the realm of mental health has been observed. Various types of universal basic income (UBI) programs have already demonstrated significant impacts on individuals’ mental health in high-income countries. Therefore, this review focuses on the situation in low- and middle-income countries. A literature review was conducted using seven electronic databases. Empirical studies of any design that implemented different types of UBI interventions in middle- and low-income countries and applied any type of mental health measures were searched for. After systematically reviewing 6822 articles, 13 empirical studies examining the relationship between various types of UBI interventions and different aspects of mental health in developing countries were identified. The collective findings of the studies suggest that UBI programs have significant positive effects on the mental health of individuals from developing countries, with the potential mediating role of unconditionality and payment frequency being noteworthy. However, these implications are limited due to the small number of studies conducted in this area and their methodological constraints. Further research with rigorous design is needed, particularly focusing on experimental control and length of follow-up periods.

## 1. Introduction

Universal basic income (UBI) is a political theory or proposal [1], representing one of the most innovative yet controversial ideas addressing poverty, unemployment, and increasing inequalities in today’s society [2,3,4,5,6,7,8]. It is a form of social welfare generally defined by five characteristics: cash payment (provided in the form of cash or cash equivalents, allowing individuals to freely dispose of the received amount); periodicity (provided at regular intervals, such as monthly or quarterly); individuality (paid to individuals); unconditionality (individuals are entitled to it without fulfilling any conditions); and universality (payment is intended for everyone, regardless of their social, employment, or economic status) [1,2,5,9,10].

While UBI resembles other social welfare programs (SWPs) in providing economic assistance to those in need, the benefits offered by most SWPs are typically only accessible to a segment of society that meets certain income or asset criteria [5]. Unconditional cash transfers (UCTs) are an exception in this regard. They are a social protection intervention addressing a key social determinant of health (income) in low- and middle-income countries, aiming to reduce poverty and vulnerabilities (e.g., orphanhood, old age, or HIV infection) [11]. They are provided without obligations and are, in that regard, similar to UBI. Both interventions are in fact so similar that some authors use them as synonyms (for example, Refs. [12,13] or see UCTs as a type of UBI intervention (for example [6])). They both share at least three (cash transfers, paid periodically, and unconditionally), often four (individuality), characteristics out of the five defined as UBI. Moreover, the vast majority of so-called “basic income” trials only partially fulfill the characteristics of UBI [14,15], which further strengthens the overlap between these two concepts. In this paper, the example of researchers such as Wilson and McDaid [16] in their solution to the dilemma of demarcating UBI and UCTs by understanding UBI as a broader concept that includes, but is not exclusively limited to UCTs, has been followed (this is also reflected in the search protocol as discussed in Materials and Methods). By doing so, it was ensured that the results of UCTs would be captured but also the results that could shed light on other characteristics of UBI that would be lost if the focus were solely on UCTs (i.e., individuality and universality). Terms such as “various types of UBI” are used while reporting on UBI and UCT studies, and the term “UBI” or “UCT” is used while reporting on individual studies, following the explicit definitions of the authors of the respective studies, whether their findings pertain to UBI or UCTs.

The proposed mental health benefits of various types of UBI are often discussed by proponents of these interventions [17]. Wealth has long been considered an important social determinant of health [18], and poverty is seen as a key driver of mental health inequities [19]. Furthermore, the principles of UBI intervention—unconditionality, individuality, and universality—are viewed as potential promoters of mental health. It is argued that since most SWPs are typically accessible only to a segment of society that meets certain income or asset criteria, recipients of such benefits are often “trapped” in poverty, as they are discouraged from earning additional income if the benefits provided by the program are reduced or discontinued when an individual’s income increases [20]. Additionally, the unconditional nature of various types of UBI is expected to help reduce the stress of recipients associated with the sanctions, strict eligibility criteria, and cumbersome bureaucracy of most SWPs [8,21,22]. The factor of individuality is seen as a measure of ensuring the social and financial independence of vulnerable household members [1,23,24]. Universality of payments is proposed to reduce poverty stigma and increase social justice [24].

Significant positive effects on mental health outcomes have been observed with various types of UBI interventions, such as reducing stress, worry, and anxiety [14,15,25], as well as lower levels of depression [25] and a lower prevalence of psychopathology both in adolescence [26] and adulthood [27], including fewer addiction symptoms [27]. Increased feelings of autonomy [28], agency [25], hope and optimism about the future [14,15], and quality of life [29] have also been reported in other studies. In the social domain, an important factor for mental health, studies have reported reduced social stigma [15,25,28], increased social integration and participation [15,25,30,31], and improved family relationships [25,32].

Upon reviewing the literature, it is evident that the understanding of how this form of economic support affects the mental health of residents remains limited. Only a handful of authors have incorporated observations regarding the direct impacts of implementing various UBI programs on residents’ mental health into their studies. This limitation is reflected in systematic and other types of review papers. A small number of reviews were found to focus on the health impact of various types of UBI as part of their broader objectives [13] or solely on health [17,33,34]. Only one review was identified that focused exclusively on mental health, but it limited the included studies to high-income countries [16].

Furthermore, the majority of programs included were only implemented in high-income countries. In fact, most studies examining the development of mental disorders have been conducted in high-income countries [35], despite low- and middle-income countries accounting for more than 85% of the world’s population [36]. This is particularly concerning given that poverty and lower socioeconomic status are often cited as major risk factors for the development of mental disorders, and mental disorders, in turn, increase the risk of poverty [37,38,39].

Limited understanding of the effects of implementing various SWPs on the mental health of residents in middle- and low-income countries represents a missed opportunity to formulate policies that could more effectively help establish and maintain the mental health of the population [12,16]. Additionally, due to the specific characteristics of UBI programs (periodicity, unconditionality, individuality, and universality), it could be inferred that compared to other SWPs, the implementation of various types of UBI programs would not yield the same outcomes in terms of the mental health of recipients [5,8,20,21,22]. Consequently, the purpose of this review is to examine the correlations between the introduction of UBI models and the mental health of residents of low- or middle-income countries. The following research objectives have been set: (a) evaluation of mental health outcomes associated with implemented programs; (b) examination of the relative impacts of the individual, unconditional, and universal principles of such programs on these outcomes; and (c) based on the analysis of the methodological characteristics of conducted studies, guidelines for further research are provided.

## 2. Materials and Methods

To ensure methodological rigor in the systematic review, adherence was given to the PRISMA 2020 guidelines [40]. The search protocol established by Wilson and McDaid [16] for their review article, which was designed to identify the mental health effects of universal basic income (UBI) in high-income countries, was followed. The effectiveness of their search strategy was demonstrated, and the current review is deemed complementary. Additionally, their approach to delineating UBI from unconditional cash transfers (UCTs) was employed. By incorporating unconditional cash transfers into the search terms, results related to UCTs were captured while preserving the broader context of UBI. This method enabled the acquisition of results that illuminated other characteristics of UBI that might be overlooked if the focus were restricted solely to UCTs (e.g., individuality and universality).

Articles were retrieved on 17 March 2023 from the Medline, Scopus, CINAHL with Full Text, APA PsycArticles, ERIC, MasterFILE Premier, and Academic Search Complete databases. Furthermore, articles were obtained through manual searches, which involved exploring the references of relevant articles, visiting key organizational websites, and reviewing the reference lists of significant journals.

Following search protocol used by Wilson and McDaid [16] for automated searching, a search string encompassing diverse terms for UBI and UCTs and terms related to mental health and a simultaneous exclusion filter that generally removes review articles were employed. Individual terms were combined using Boolean operators (AND, OR, and NOT), resulting in the following search string: (“Universal Basic Income” OR “Basic Income” OR “Citizens Income” OR “Citizens Basic Income” OR “Basic Income Guarantee” OR “Basic Living Stipend” OR “Guaranteed Annual Income” OR “Income Maintenance” OR “Universal Income Guarantee” OR “Unconditional Cash Transfers”) AND (“Mental Health” OR “Mental Disorder” OR “Mental Illness” OR Well-being OR Depression OR Anxiety OR “Life Satisfaction” OR “Quality of Life” OR “Psychological Distress”) NOT review. An English language search filter was applied to all databases.

Modifying the search protocol used by Wilson and McDaid [16] with regard to point “c” (see below), inclusion criteria required that (a) the study includes at least one outcome (measurement) related to a specific aspect of mental health, (b) it involves the application of an experimental intervention of UBI or UCT, which must be necessarily periodic and in the form of a cash payment, and additionally meets one of the following three criteria of UBI [2]: (i) individuality—paid to individuals (as opposed to households); (ii) it is universal, meaning it is paid based on residency within the country; and/or (iii) unconditional—there is no income threshold to receive the money, and individuals do not need to perform any conditional work to obtain it; and (c) the study was conducted in a country classified as low- or middle-income by the World Bank [41].

Articles were initially evaluated based on titles and abstracts during the selection process and subsequently based on the full text of the articles. Articles that did not meet the inclusion criteria were excluded. Articles were retrieved from the databases by one author, while the suitability of individual articles for inclusion was assessed by multiple authors, with each article evaluated by one author. Data from studies meeting the inclusion criteria were collected by multiple authors for each article. The collected data included: authors’ names and publication year, name or type of applied UBI intervention, study design, sample and control group information, measurement/outcome of mental health, results, and methodological limitations.

The quality of each study was evaluated based on the Critical Appraisal Skills Programme (CASP) checklists [42]. To evaluate the quality of each study, a checklist corresponding to the study design was used, with each checklist comprising eleven questions addressing various aspects: validity, methodological adequacy, reporting of results, and their applicability.

## 3. Results

**Figure 1:**
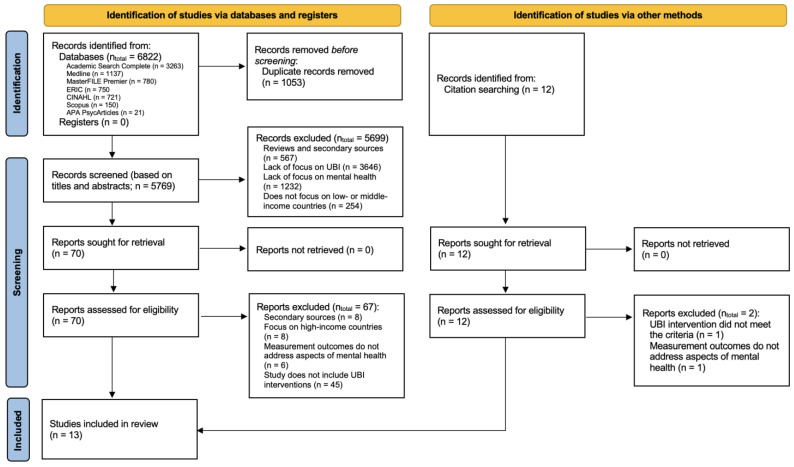
Prisma flow chart of article identification and selection.

In total, 6822 articles were obtained from the databases, and after removing duplicates, 5769 remained. Following exclusion based on titles and abstracts, and subsequently based on the full texts, three articles were retained for inclusion. Through manual searching via citation tracking in published scholarly articles, twelve articles were identified as potentially relevant. Of these, ten remained for inclusion based on the evaluation of the full articles. The entire process of article identification and selection is depicted in Figure 1.

The included articles in the review are presented in Table 1. The covered studies range in publication dates from 2013 to 2020. Eleven out of thirteen included studies were designed as randomized controlled trials (RCTs), while only two were of a longitudinal type with qualitative and quantitative methodology. Four studies focused exclusively on adolescents (aged 12 to 29), one exclusively on elderly citizens (aged 70 to 74), one included both parents and their children, and the remaining seven studies were on adults in general. The included studies were further categorized by the country where the intervention was applied and additionally (substructured) by the program implemented within the country. The included countries corresponding to the individual included articles are as follows: Malawi, Zambia, Kenya, Mexico, and Ghana. Among these, Malawi and Zambia belong to low-income countries, while Kenya, Mexico, and Ghana belong to middle-income countries [41].

The quality assessments of individual included articles are displayed in Table 2. According to the two types of methodological designs, the CASP appraisal scale for randomized controlled trials and qualitative studies was accordingly utilized. Based on the scale results, it was found that all studies achieved a moderate to high level of quality.

All reviewed programs provided unconditional cash payments, with six of them being distributed on an individual basis. However, the principle of universality was disregarded in all cases, as the programs targeted specific economic groups. Because the included UBI programs did not fulfill all the characteristics of UBI and were mostly not experimentally controlled, it was difficult to distinguish between the general effects of various types of UBI programs and the specific effects of UBI characteristics. In the following subsections, the results are presented according to aspects of mental health that were analyzed in the reviewed articles. First, the results related to the broader topic of general mental health and psychological well-being are presented, then the connections with depression and stress within this framework are examined. Within each subsection, the discovered links with the general effects of various types of UBI programs are identified first, followed by the potential connections between the aspects of mental health and individual characteristics of UBI.

### 3.1. General Mental Health and Psychological Well-Being

General mental health and psychological well-being were measured in seven studies using instruments that assessed overall health. These included the General Health Questionnaire (GHQ-12) [45], the Perceived Stress Scale (PSS) [51,52,53], and different adapted, extended, or shortened versions of the World Health Organization Quality of Life questionnaire [61]. In the study by Natali et al. [45], the WHOQOL included five questions inquiring about participants’ satisfaction with their child’s life, future, health, happiness, and general life satisfaction, as well as a question about the overall happiness of the parents. Kilburn et al. [47] examined carers’ SWB through their Quality of Life scale, which they constructed from questions drawn from the SWLS and WHOQOL, with the addition of a relative well-being question. Similarly, Handa et al. [50] also used the WHOQOL, but only the “positive feeling” and “overall life and health” domains, and additionally, the Mental Health Inventory (MHI-5) was utilized by Baird et al. [46]. All these studies consistently reported improved psychological well-being and mental health in various UBI programs.

Recipients of unconditional cash transfers in the GiveDirectly UBI program [51,52,53] showed increased psychological well-being compared to the control group, attributed to reductions in stress and depressive symptoms and increases in self-reported life satisfaction and happiness. These results were observed both within and between village analyses [51,52,53]. Furthermore, the Child Grant Program [45] investigated whether unconditional periodic cash transfers affected the self-rated happiness of women (mothers of children under 5 years) from poor households. The program was particularly effective in improving the subjective well-being of recipients, their assessments of their children’s satisfaction and health, and their positive outlook on the future. After 36 months, a significant increase was observed in the proportion of women who felt happy and generally more satisfied with life. After 48 months, the effect of unconditional cash transfers on happiness and satisfaction persisted and even increased. Similarly, Daidone et al. [55] measured happiness using a simple question, “Are you happy with your life?”, in the Livelihood Empowerment against Poverty Program, aimed at households from three regions in Ghana (Brong-Ahafo, Central, and Volta). This program lasted 24 months and showed an increased likelihood of happiness in the intervention group by 16 percentage points compared to the control group.

Baird et al. [46] also examined the spillover effect and the impact of the amount of unconditional cash transfers on the psychological distress of girls. Regarding the spillover effect, it was found that girls who did not live in the same household as the cash transfer recipients had statistically significantly higher levels of psychological distress. These differences were not present when non-recipients lived in the same household as the recipients, suggesting that cohabitation provided similar psychological benefits. The spillover effect on psychological distress remained statistically significant when the two groups were evaluated together. These significant differences were present only during the period of receiving transfers—after the program ended, there were no differences in psychological distress. Concerning the amount of cash transfers, it was found that receiving the minimum amount statistically significantly reduced psychological distress compared to the control group. Psychological distress increased significantly with each additional dollar received under conditional cash transfers for the parents of the girls. In the case of unconditional and conditional cash transfers, there were no statistically significant differences, but there was a statistically significant reduction in psychological distress after the program ended, indicating long-term effects. These findings suggest that when transfers are received by parents as a conditional source of income for the entire family, dependent on the girl’s behavior, it can be burdensome, causing greater psychological distress.

The aspect of unconditionality was examined in the Randomized Cash Transfer program [46], where one group of schoolgirls received cash payments conditional on over 80% school attendance, while another group received payments regardless of attendance (unconditional). The program had a significant positive effect on mental health, with a notable reduction in psychological distress in both groups. However, the effect was significantly greater in the group receiving unconditional payments. The authors concluded that the conditional cash transfers burdened the girls with efforts and concerns about meeting the conditions to retain the payments. The program did not affect the mental health of girls who had dropped out of school. Other studies did not precisely measure the impact of the unconditionality aspect of programs or included control groups with conditional payments.

The impact of the periodicity of unconditional cash transfers was examined in the GiveDirectly UBI program [51,52,53]. The effects of monthly versus lump-sum payments were compared, but no differences were found between payment methods in relation to general mental health. However, the authors [52,53] observed that lump-sum payments were associated with lower cortisol levels but not with other aspects of psychological well-being. On the other hand, monthly payments more significantly increased food security, which was strongly associated with psychological well-being. It is suggested that higher cortisol levels in monthly payments may result from waiting for payments, less flexibility in money management, and related stress. Based on the results regarding the impact of payment periodicity on general mental health and psychological well-being and the lack of additional research in this area, it can be concluded that the periodicity and lump-sum nature of cash payments potentially relate to improvements in different specific segments of psychological well-being (such as cortisol levels and food security), while there is currently no evidence linking this factor to general mental health.

### 3.2. Depression

Depression was measured in nine studies, eight of which used the Center for Epidemiologic Studies Depression Scale (CES-D) [43,44,49,50,51,52,53,54] and one using the Geriatric Depression Scale [48]. Seven studies found a reduction in depressive symptoms [48,49,51,52,53,54], while two did not observe these effects [43,44].

In a studied Kenyan program, the effects of intervention on depression in adolescents were observed [49]. A comparison between the control and intervention groups revealed a lower level of depressive symptoms in the intervention group, which was statistically significant only for young males. In young females, the trend was the opposite—after receiving cash transfers, young females showed an increase in depressive symptoms. An interesting perspective can be seen in the study by Handa et al. [50], where depression among young people was studied as a function of parents’ SWB—the researchers found that unconditional cash transfers improve parents’ quality of life and future expectations, which in turn has an impact on reducing depressive symptoms in their children.

Unconditional cash transfers were also included in the Multicategory Targeted Grants (MCTG) program, which showed no effects on depression levels at both 24 [43] and 36 months after the program’s start [44]. A key difference in the program execution between Kilburn et al. [49] and MCTG was that in the MCTG program, cash transfers were given to entire households, not accounting for the individuality aspect of UBI. In contrast, Kilburn et al. [49] provided cash transfers to individuals, which might explain the lack of a connection with depression in the MCTG program.

Both individuality and unconditionality were considered in the 70 y más program [48], where a significant reduction in depressive symptoms among the elderly was found. Conversely, the individuality aspect of unconditional cash transfers was not considered in the GiveDirectly UBI program [51,52,53]. However, after three years of the program [51], significantly lower levels of depression were observed in households receiving unconditional cash transfers compared to “spillover” households within the same village.

The periodicity aspect of unconditional cash transfers was considered in the GiveDirectly UBI program as part of the research by Haushofer and Shapiro [51,52,53] and Banerjee et al. [54]. Monthly payments were found to be more effective in reducing depressive symptoms compared to lump-sum payments at the program’s start, where such effects were not observed. In the study by Salinas-Rodriguez et al. [48], a significant effect of anticipation was also identified. This effect led to increased depression among older adults who were not included in the program due to not meeting the minimum age requirement of 70 years.

### 3.3. Stress

Of the 13 reviewed studies, four measured changes in stress levels associated with UBI programs. Haushofer and Shapiro [51,52,53] used the Perceived Stress Scale (PSS) and cortisol measurements, while Baird et al. [46] used the General Health Questionnaire 12 (GHQ-12). All these studies observed a reduction in stress with the receiving of unconditional cash transfers.

Haushofer and Shapiro [51,52,53] included both perceived stress and cortisol measurements in their research. While participants in households receiving unconditional cash transfers consistently reported lower perceived stress levels compared to those not receiving cash transfers, no significant differences were found in physiologically measured cortisol levels. The authors explained these conflicting results by suggesting that cortisol is harder to influence and measure or that self-reporting led to significant discrepancies due to the experimental demand effect. Furthermore, differences in measurements based on the gender of the recipient in the household receiving cash transfers were examined. It was found that in households where women received the transfers, both partners exhibited lower cortisol levels compared to households where men received the transfers. Additionally, within households where women received the transfers, women showed even lower cortisol levels compared to men. The authors proposed potential explanations such as female empowerment and higher self-esteem or that men felt less stress and responsibility for the family’s livelihood when women received the cash transfers instead of them.

Regarding the periodicity of unconditional cash transfers, individuals in households receiving monthly transfers, despite reporting reduced stress, still showed a trend of higher cortisol levels compared to those receiving lump-sum payments. This could be due to higher stress related to difficulties in saving, budgeting, and managing expenses among household members receiving monthly transfers [52,53]. The included studies do not provide insights into how the individuality aspect of transfers influences changes in stress levels.

### 3.4. Optimism and Hope

Optimism was measured by Haushofer and Shapiro [51,52,53] using a revised form of Scheier’s Life Orientation Test [59], while Kilburn et al. [48] and Handa et al. [49] measured hope using the Children’s Hope Scale [62]. Daidone et al. [55] reported hope emerged as one of the themes in the qualitative part of their study.

In their first analysis, Haushofer and Shapiro [53] did not find statistically significant changes in optimism among unconditional cash transfer recipients compared to the control group, but they did observe a statistically significant increase in optimism within the spillover effect group. There were also no differences based on gender, monthly versus lump-sum payments, or larger versus smaller transfers. In their second analysis, the authors found that optimism was statistically significantly higher in households receiving cash transfers compared to the control group, but there were no statistically significant differences in other cases [52]. As for long-term effects, there were no changes in optimism, except for a statistically significant decrease in optimism within the spillover effect group [51].

Kilburn et al. [49] found that unconditional cash transfer recipients had a statistically significantly higher prevalence of hope and optimism compared to the control group. Additionally, they found that men who received cash transfers had statistically significantly higher odds of achieving scores above the median on the hope scale compared to those who did not receive transfers. There were no statistically significant differences among women. Handa et al. [50] examined hope among young people as a function of parents’ SWB and found consequential relationships between the variables—unconditional cash transfers improve parents’ quality of life and future expectations, which in turn increase their children’s hope. Improved levels of hope in the intervention group after two years of the program’s duration were also reported by Daidone et al. [55], but this topic was only included in the qualitative part of their study.

### 3.5. Locus of Control

Locus of control was measured in the studies by Haushofer and Shapiro [51,52,53] and Banerjee et al. [54]. In the former studies, the Locus of Control Scale [60] was used, while in the latter study, the authors did not specify the measurement instrument. No statistically significant changes in locus of control were observed in these studies, except for the long-term effects or payments in the study by Haushofer and Shapiro [51].

In the short-term studies by Haushofer and Shapiro [52,53], no statistically significant differences in locus of control were observed between individuals in households receiving cash transfers and those not receiving them, nor were there any differences based on gender. Furthermore, there were no differences between monthly cash payments and lump-sum payments, and the spillover effect did not impact changes in locus of control. However, a statistically significant change in locus of control was observed as a long-term effect of unconditional cash transfers; Haushofer and Shapiro [51] found that internal locus of control significantly increased among cash transfer recipients. Concurrently, they found that the spillover effect also had a statistically significant change, with households that replaced their thatched roofs with metal ones experiencing an increase in external locus of control, albeit to a negligible extent. Banerjee et al. [54] did not find any statistically significant differences in locus of control in any case, either short-term or long-term.

### 3.6. Other Parameters

Under this section, all those psychological constructs or parameters for which the authors did not use established psychometric tests are considered. Consequently, this includes expectations for a better future life [43,44,47,50], empowerment [48], the Women’s Empowerment Index [51,52,53], worries [51,52,53], and self-esteem [55]. Expectations were measured with simple questions [43,44,47,50], e.g., “Do you think your life will be better, the same or worse in one year/three years/five years from now?”, and empowerment was assessed through the subjective ability to participate in household decision making [48]. Haushofer and Shapiro [51,52,53] calculated the Women’s Empowerment Index based on the standardized average of the violence index and the relationship index, while worries were measured using a custom-designed questionnaire.

Regarding expectations, Handa et al. [50] and Kilburn et al. [47] found that participants were significantly more likely to believe that their lives would improve in the future after the intervention. A similar trend was also observed in the AIR study [43,44], but the results were not statistically significant.

Salinas-Rodriguez et al. [48] found that the program significantly increased the empowerment of older adults in terms of participating in non-economic household decisions as well as overall involvement in important household decisions. Individuals who had not yet received cash transfers due to being too young significantly increased their participation in household economic decisions, while there was no significant increase in empowerment for non-economic decisions. This indicates an expectation effect but only in the case of having financial resources.

In terms of the short-term effects of the UBI program on the Women’s Empowerment Index, Haushofer and Shapiro [52,53] found no overall changes. A statistically significant difference was only observed in the case of the spillover effect and the amount of cash transfers; women’s empowerment increased among those who saw others receiving cash transfers. Additionally, empowerment increased among women in households receiving larger cash transfers compared to those with smaller transfers. There were no long-term changes in women’s empowerment, except for a statistically significant increase in empowerment within the spillover effect, but this was exploratory in nature with *p* < 0.100 [51].

Haushofer and Shapiro [53] found in their first analysis that there were no statistically significant differences regarding worries, except by the gender of the recipient, with statistically significantly lower levels of worry in households where the female received the transfers, although at *p* < 0.100. The second analysis showed that cash transfer recipients had significantly reduced worries compared to the control group, while there were no other differences in worries [52]. Haushofer and Shapiro [51] did not explicitly report on the long-term effects of the UBI program on the presence of worries in their article. Daidone et al. [55] reported improved levels of self-esteem in the intervention group after two years of the program, though this topic was only addressed in the qualitative part of their study.

The mentioned findings primarily indicate the impact of the UBI program on the empowerment of older adults, while changes in other cases are inconsistent or exploratory in nature. In all cases, non-standardized methods of obtaining information were used, so further research could enhance the existing literature by studying the impact of UBI programs on constructs using established instruments.

## 4. Discussion

The purpose of this study was to review research on various types of universal basic income (UBI) programs in low- and middle-income countries [2], with a focus on indicators of mental health. The aim was to determine the impacts of various UBI programs on mental health and to assess the effects of various UBI characteristics (periodicity, unconditionality, individuality, and universality) on mental health outcomes. Additionally, guidelines for future research were provided based on an analysis of the methodological characteristics of the studies reviewed. The following discussion is organized to address these research questions.

### 4.1. Overall Effect of Various UBI Programs on Different Aspects of Mental Health

Poverty is one of the most significant social issues and is negatively associated with various aspects of mental health among vulnerable groups, further contributing to its socioeconomic burden [39,63,64]. This supports the argument that mental health should be a crucial aspect of development strategies implemented by governments, non-governmental organizations, bilateral and multilateral agencies, global partnerships, private foundations, and other interest groups [64]. This systematic review implies that in low- and middle-income countries, various types of UBI could be a significant part of these development strategies. The aggregate findings from the reviewed studies suggest that the implementation of various types of UBI programs in low- and middle-income countries can lead to improvements in overall mental health and increased happiness [51,52,53,55], life satisfaction [51,52,53], and well-being [45,51,52,53], as well as reductions in stress levels [52,53]. Significant reductions in distress and the likelihood of mental disorders were observed in young girls, though this effect was noted only after 12 months and not after 24 months of program implementation [46]. Higher levels of hope were reported in young men [49], and improvements in parents’ quality of life and future expectations were also noted [47,50], which in turn alleviated depression and increased hope in children [50]. Qualitative studies further reported positive effects on self-esteem, hope, and overall happiness among participants [55].

The impact of various UBI programs on depression levels, worries, feelings of empowerment, optimism, hope, and locus of control remains unclear. Although some findings are inconclusive, and others are only exploratory, further exploration of the potential positive effects is warranted in future research. Regarding depression, more studies have found significant reductions in depression levels [48,49,51,52,53,54] compared to those that found no reduction [43,44] in the treatment group relative to the control group.

Studies on worries are also inconclusive and exploratory in nature. One study did not find any statistically significant differences regarding worries except for a very small effect of the recipient’s gender [53]. Another study found that cash transfer recipients had significantly reduced worries compared to the control group, but there were no other differences in worries [52].

The limited body of research on feelings of empowerment found that the non-contributory pension program significantly increased the empowerment of older adults in terms of participating in non-economic household decisions as well as overall involvement in important household decisions [48]. Surprisingly, individuals who had not yet received cash transfers due to being too young also significantly increased their participation in household economic decisions, but there was no significant increase in empowerment for non-economic decisions. Regarding women’s empowerment, studies found no change in the overall Women’s Empowerment Index in females receiving unconditional cash transfers, either in the short term [52,53] or long term [51]. However, there are some interesting findings, currently only exploratory, that could prove to be important in the future. Namely, empowerment increased among women in households receiving larger cash transfers compared to those with smaller transfers [53], and women’s empowerment increased among those who saw others receiving cash transfers while they alone had not received funds [51,53].

Studies regarding the effect of various UBI programs on optimism, hope, and expectations about the future are also inconsistent. Receiving cash transfers from various UBI programs appears to increase optimism [49,52], with participants significantly more likely to believe that their lives would improve in the future after the intervention [47,50]. However, some studies fail to identify a statistically significant effect [43,44], and others found that optimism does not change in the long term [51]. The spillover effect on optimism is particularly intriguing. Short-term optimism seems to increase among individuals who do not receive the cash themselves but observe others receiving it, whereas it decreases in the long term. One interpretation is that individuals who see others receiving cash become optimistic because they believe it will benefit the broader environment they live in or expect it will also affect them, either directly (receiving payments) or indirectly (improving living conditions due to the recipients).

At this point, the results concerning the effect of various UBI transfers on locus of control remain preliminary. Banerjee et al. [54] did not find statistically significant changes in locus of control in either the short term or long term. Haushofer and Shapiro [51] found long-term effects in two conditions, but the effects were so small as to be negligible. However, it is worth highlighting the diverse direction of changes in locus of control: the unconditional cash transfer group experienced a more internal locus of control, while the spillover effect group (families living in the same village as those receiving unconditional cash transfers) experienced a more external locus of control. Thus, those who received cash transfers felt they had more control over their lives in some way, while individuals in the spillover effect group felt that outcomes were outside their control.

This could be interpreted to mean that cash transfer recipients felt more empowered due to the received money, while the spillover effect group felt that things in their environment were left to an external control since they did not receive payments themselves but observed others receiving them. This interpretation aligns with the self-serving bias, where individuals attribute good or desired events to their own control while blaming external factors for undesired events [65]. It is possible that recipients attributed their receipt of funds to their own merit, while individuals within the spillover effect group attributed control to external influences since they did not receive the cash payments while observing others around them receiving them.

### 4.2. The Effect of UBI Characteristics on Mental Health Outcomes

The importance of UBI characteristics mostly remains unclear, as the majority of the reviewed studies either did not consider these factors or did not experimentally control for them.

Regarding unconditionality, all of the studies included in the review provided cash transfers on an unconditional basis, but only one study [46] controlled this aspect experimentally. In this study, one group of schoolgirls received cash payments conditional on over 80% school attendance, while another group received payments regardless of attendance (unconditional). In both groups, there was a notable reduction in psychological distress, but the effect was significantly greater in the group receiving unconditional payments. Furthermore, psychological distress increased significantly with each additional dollar received under conditional cash transfers. The authors concluded that the conditional cash transfers burdened the girls with efforts and concerns about meeting the conditions to retain the payments. This suggests that the unconditionality of payments likely plays a significant mediating role in the impact of interventions on mental health.

This conclusion aligns with the findings of Wilson and McDaid [16], who, while reviewing UBI studies in high-income countries, found a similar trend. They state on page 11 that “studies consistently reported clear and significant improvement in mental health when the conditionality associated with traditional welfare was removed or replaced with more supportive, tailored interventions”.

The periodicity aspect of UBI programs was considered in the GiveDirectly UBI program as part of the research by Haushofer and Shapiro [51,52,53] and Banerjee et al. [54], where the effects of monthly versus lump-sum payments were compared. Although no differences were found between these two payment methods in relation to general mental health, monthly payments significantly increased food security, which was strongly associated with psychological well-being. Monthly payments were also found to be more effective in reducing depressive symptoms and stress levels compared to lump-sum payments at the program’s start, where such effects were not observed. However, individuals in households receiving monthly transfers, despite reporting reduced stress, still showed a trend of higher cortisol levels compared to those receiving lump-sum payments [52,53]. This could be due to higher stress related to difficulties in saving, budgeting, and managing expenses among household members receiving monthly transfers [52,53].

Research does not provide insight into the importance of individuality (six programs included in our review made individual payments, but none compared this to payments on a family basis) and universality roles (no pilot implemented universality in the pure sense of the word). Regarding individuality, although studies showed a positive impact of interventions on the general mental health of recipients, whether payments were made on an individual basis (e.g., [46]) or not (e.g., [45,47,50,55]), reliable insight is still lacking due to the absence of controlled comparisons of these effects within individual studies. However, some hypotheses on its potential effect on depression levels might be gained by comparing the results of Kilburn et al. [49] and AIR [43,44]. A key difference in program execution between Kilburn et al. [49] and the MCTG [43,44] was that in the MCTG program, unconditional cash transfers were given to entire households, not accounting for the individuality aspect of UBI. In contrast, the analysis by Kilburn et al. [49] provided cash transfers to individuals, which might explain the lack of a connection with depression in the MCTG program.

### 4.3. Other Lines of Inquiry

Although not directly related to the research goals, some interesting observations that deserve further attention from researchers were identified due to their potential impact on mental health in different types of UBI schemes.

First, the identified link is between the indirect effects of UBI interventions for parents and the simultaneous mental health benefits these interventions provide to their children. As emphasized by Wilson and McDaid [16] in their review of the mental health effects of UBI pilots, many of the studies conducted in high-income countries show a significant increase in the recipients’ ability to invest time in improving relationships with family and friends. Among others, the Great Smoky Mountains Study [27,66] showed significant and long-lasting improved mental health outcomes in children when their parents were provided with unconditional, individual payments. Similarly, the study by Handa et al. [50], included in this review, found that unconditional cash transfers improved parents’ quality of life and future expectations, which in turn reduced depressive symptoms and increased hope in their children. Though not directly focusing on parent–child relations, the study by Baird et al. [46] examined the spillover effect and the impact of the amount of unconditional cash transfers on the psychological distress of girls. Regarding the spillover effect, it was found that girls who did not live in the same household as the cash transfer recipients had statistically significantly higher levels of psychological distress. However, these differences were not present when non-recipients lived in the same household as the recipients, suggesting that cohabitation provided similar psychological benefits. These studies imply that the mental health benefits extend beyond only the individual recipients of various types of UBI transfers, which is encouraging in light of the difficulties in implementing these programs on a larger, even universal, scale.

A second line of potential inquiry is related to different mental health outcomes depending on the gender of the recipient of intervention. In a studied Kenyan program, the effects of intervention on depression in adolescents were observed [49]. A comparison between the control and intervention groups revealed a lower level of depressive symptoms in the intervention group, which was statistically significant only for young males. In young females, the trend was the opposite—after receiving cash transfers, young females showed an increase in depressive symptoms. The authors interpreted this as a result of the generally higher and more persistent depressive symptoms among young females, which are not solely influenced by economic conditions. Ngcobo and Pillay [67] commented on this, identifying domestic violence, unemployment, sexual violence, relationship problems, and criminal experiences as primary reasons for depression among African women seeking psychological help. Most women reported having no one in their social circle to turn to with their problems—factors not addressed by various UBI programs.

Haushofer and Shapiro [53] found in their first analysis that there were no statistically significant differences regarding worries, except by gender of the recipient, with statistically significantly lower levels of worry in households where the female received the transfers, although at *p* < 0.100. Additional research [51,52,53] found that in households where women received the transfers, both partners exhibited lower cortisol levels compared to households where men received the transfers. Additionally, within households where women received the transfers, women showed even lower cortisol levels compared to men. The authors proposed potential explanations such as female empowerment and higher self-esteem or that men felt less stress and responsibility for the family’s livelihood when women received the unconditional cash transfers instead of them. Similarly, Pereira et al. [68] reported higher well-being, reduced stress, and overall positive effects of unconditional cash transfers from the male perspective in the family when women received the transfers. Another study [69] reported reduced stress and increased female empowerment in similar programs where women were the recipients of cash transfers. However, these two studies [68,69] did not examine and compare the effects on both women and men simultaneously, nor did they investigate the impact when men were the recipients of cash transfers in the family, limiting the implications of their results. Nonetheless, these studies support the findings of Haushofer and Shapiro [51,52,53], generally indicating that cortisol plays a significant role in explaining the reduction in subjectively perceived stress associated with cash transfers only in households where women receive the transfers.

### 4.4. Limitations and Ideas for Further Research

The presented study is, to our knowledge, the first review article summarizing the link between the impacts of various types of UBI in middle- and low-income countries and different aspects of mental health. In the review, seven databases were covered, and initially, 6822 articles were identified, with the final selection including 13 empirical articles.

It is important to note the various limitations resulting from the design of this study or representing the methodological constraints of the studies included in this review. A common limitation of all included studies is that self-reporting was used for mental health indicators. Although the questionnaires employed were validated for their suitability, reliance was placed on self-reported data. One of the limitations of this review is the reliance solely on a systematic literature review rather than a meta-analysis of the studies. The studies included were highly diverse, employing a wide range of instruments to measure aspects of mental health, and various statistical methods were used for data analysis. Consequently, reliance was placed on the reliability and validity of the results from the included studies, an issue that could potentially have been mitigated by analyzing all data using a single, rigorous methodology.

The present review contains a fairly small final selection of studies that included different types of UBI interventions and used different questionnaires to measure impacts on the same mental health outcomes, reducing the comparability of results. Additionally, only studies published in English were included in the review. The questionnaires used were also only partially standardized [47,51,52,53] or entirely non-standardized [43,44]. In Banerjee et al. [54], the measurement instruments used were poorly specified, and in Daidone et al. [55] and Salinas-Rodriguez et al. [48], the qualitative part used questions in semi-structured interviews, limiting the validity of the results.

The effects were examined over different periods in the studies, with the longest being 48 months [45,50], but there is a possibility that certain effects may occur later, especially in studies that assessed effects after only 12 months [48] or after 17 months [47]. Different studies used different threshold values for the same questionnaire [43,44,54] or used different versions of the same questionnaire (e.g., CES-D with 10 items in AIR [43,44] and Kilburn et al. [49], with 20 items in Haushofer and Shapiro [51,52,53] and Banerjee et al. [54], and ten selected questions from CES-D-20 in Handa et al. [50]), limiting the comparability of their results within the present analysis.

Authors of the included studies also report other methodological limitations, such as using certain questionnaires only at the second of two measurements conducted to assess intervention effects [46], adding participants to the control group later [53], a large sample attrition [54], and Kilburn et al. [49] did not have baseline data in their study, so their findings were based solely on comparing results between the experimental and control groups at the final measurement. Daidone et al. [55] note more than half the control households lived in different regions compared to intervention households which could have an impact on the results. Kilburn et al. [47] warn that the positive time trend in their study is an anomaly. The authors note that data collection was conducted during both the dry and rainy seasons; there might be a connection between rain and subjective well-being.

The limitations of certain studies may also include the potential spillover effect of certain resources within the village, which could lead to inaccurate results. This effect was only considered in the studies by Haushofer and Shapiro [51,52,53], while in other studies, it was mentioned only as a possible phenomenon in the introduction [43,44,46,47,54]. In the introduction, Handa et al. [50] briefly mentioned the spillover effect in the parent–child relationship, specifically how life events impacting parents influence the mental health and well-being of children. Interestingly, in the results section, the authors did not discuss the spillover effect, despite the analyses being closely related to this concept—they examined the impact of changes in parents’ well-being, caused by income shock, on changes in children’s well-being.

In Banerjee et al. [54], the intervention was implemented during an epidemic, which could have been a disruptive factor in measuring mental health and consequently hindered the generalizability of their results to non-epidemic times. Lastly, certain studies were conducted in remote areas, such as the AIR study [43,44], which was conducted in the most remote parts of Zambia with limited access to paved roads leading to the capital city. As a result, participants had limited access to services such as healthcare and education, which could limit the program’s ability to impact these two areas and consequently affect their mental health. Nevertheless, the quality of the reviewed studies, based on the CASP appraisal checklist [42], is relatively high, as all scored eight or above on an 11-point scale (for RCTs) or seven or above on a 10-point scale (for qualitative studies), except for two studies on the GiveDirectly UBI program [51,53].

Based on the findings and the mentioned limitations, it is recommended for future research to conduct more studies on the effects of various UBI on individuals’ mental health in middle- or low-income countries, as, in terms of the number of studies, high-income countries are still more researched [16]. It would also make sense to design studies in a way that allows for a direct examination of the impact of different characteristics of UBI on mental health. For example, although a trend has been identified, more research is needed to draw solid conclusions about the effect of various UBI programs on depression levels. Future studies examining the impact of UBI on optimism and hope should focus on both short-term and long-term effects and aim to unify measurement instruments. Additionally, more comparable studies on the effects of various types of UBI programs on locus of control are needed, and more robust measurements of locus of control should be used. It would also be very useful for future studies to verify quantitatively what the authors of the included studies have found using qualitative methodology. By employing quantitative methodologies to corroborate qualitative insights, researchers could provide a more comprehensive and nuanced understanding of the effects of UBI programs—such an approach would help to validate and potentially generalize qualitative observations, thereby strengthening the overall evidence base. This combined methodological approach could lead to more robust and conclusive findings, offering clearer implications for the design and implementation of UBI programs and their impact on various mental health outcomes.

Some of the reviewed studies [46,51,52,53] have already examined the characteristics of the unconditionality, individuality, and periodicity of UBI, but greater systematicity is needed, particularly in terms of comparing groups that consider a certain aspect of UBI with those that do not. It would be useful to conduct more follow-up assessments of studies after several years, to check both the longevity of effects and to capture those that may potentially occur with a delay. It is also believed that further research is important to explore the feasibility of incorporating additional aspects of UBI into research on the effects of UBI, such as transfer amounts, based on the findings of Baird et al. [46], which showed a statistically significant influence of UBI amounts on mental health. Research investigating the connection between psychological effects and biological markers, such as cortisol, would also be welcome, as well as research into trends identified in the included studies.

It is especially recommended that further exploration of the impact of various types of UBI on mental health for both genders depending on whether the money is received by a man or a woman be conducted, as the findings in this area are inconsistent [52,53]. It is also suggested that mediating factors both in the aforementioned gender context and in the context of the conditionality of cash transfers, which has been found to be significantly less effective in reducing stress and distress for girls than unconditional transfers [46], be explored. Finally, it would be beneficial to conduct a comprehensive meta-analysis, which could enhance the validity of studies and consequently allow for the generalization of findings. This approach could yield more precise evidence regarding trends across multiple studies related to UBI in low- and middle-income countries. Such evidence could improve the understanding of the effects of cash transfers and aid in the planning of further, more effective interventions.

## Figures and Tables

**Table 1:** Characteristics of individual included studies.

Authors	Intervention	Country	Periodic Cash Transfers	Individuality of Intervention	Universality of Intervention	Unconditionality of Intervention	Study Design	Sample	Control Group	Measurement of Mental Health	Results
American Research Institute [43]	Multicategory Targeted Grants	Zambia	✓	X	X	✓	Randomized controlled trial (RCT)	*N* = 844; adolescents (aged 15–19)	*N* = 922; adolescents (aged 15–19)	CES-D-10 ^a^	Intervention did not significantly reduce depression levels after 24 months.
American Research Institute [44]	Multicategory Targeted Grants	Zambia	✓	X	X	✓	RCT	*N* = 1040; adolescents (aged 16–23)	*N* = 1177; adolescents (aged 16–23)	CES-D-10	Intervention did not significantly reduce depression levels after 36 months.
Natali et al. [45]	Child Grants Program	Zambia	✓	X	X	✓	RCT	*N* = 1084; women (M = 29 years)	*N* = 1119; women (M = 29 years)	Adapted and supplemented WHOQOL questionnaire	Significantly increased subjective well-being in the treatment group after 36 and 48 months.
Baird et al. [46]	Randomized Cash Transfer	Malawi	✓	✓	X	✓ (as a condition of the study)	RCT	*N* = 1225; unmarried girls, grouped by schooling (aged 13–22)	*N* = 2574; unmarried girls, grouped by schooling (aged 13–22)	GHQ-12 ^b^, MHI-5 ^c^	Significant reduction in psychological distress and likelihood of mental disorders in school-going girls after 12 months but not after 24 months. There were no significant differences in those who had dropped out of school during the intervention.
Kilburn et al. [47]	Malawi’s social cash transfer program	Malawi	✓	X	X	✓	RCT	*N* = 1678 households	*N* = 1852 households	Measuring subjective well-being via Quality of Life scale (questions drawn from SWLS and WHOQOL), future expectations questions, and relative well-being question	Strong positive impacts of the income shock on caregivers’ quality of life and their perception of future well-being; no impact on their perception of relative well-being.
Salinas-Rodriguez et al. [48]	Program 70 y más (non-contributory pension program)	Mexico	✓	✓	X	✓	Qualitative and quantitative study	*N* = 1353; elderly from areas with 2500 or fewer residents (aged 70–74)	First control group for direct comparison: *N* = 888; elderly from areas with more than 2500 residents (aged 70–74); Second control group for expectation effect: *N* = 2227; elderly (aged 65–69)	GDS ^d^, questions regarding individuals’ ability to participate in important non-economic and economic household decisions, semi-structured interview with questions such as “Do your children tell you how to spend your money?” and “How do you feel now that you are receiving the Program?”	Significant reduction in depressive symptoms and increase in empowerment in the intervention condition.
Kilburn et al. [49]	Cash transfer program for orphans and vulnerable children	Kenya	✓	X	X	✓	RCT	*N* = 1408; adolescents (aged 15–24)	*N* = 598; adolescents (aged 15–24)	CES-D-10, State Hope Scale	After 4 years of receiving cash transfers, young men had significantly fewer depressive symptoms and higher levels of hope compared to the previous year and compared to the control group.
Handa et al. [50]	Cash transfer program for orphans and vulnerable children	Kenya	✓	X	X	✓	RCT	*N* = 1266 households	*N* = 545 households	Parents’ subjective wellbeing: WHOQOL domains “positive feeling” and “overall life and health” and three subjective future well-being questions; Children’s subjective wellbeing: 10 selected questions from CES-D-20 and Children’s Hope Scale	A positive income shock improves the quality of life and future expectations of parents, which in turn alleviate depression and increase hope in children.
Haushofer & Shapiro [51]	GiveDirectly UBI program	Kenya	Both: periodic and lump-sum payments	✓	X	✓	RCT	*N* = 471 households	*N* = 505 households (‘spillover’ ^e^ control); *N* = 852 households (pure control)	CES-D-20 ^f^, PSS ^g^, questions on happiness and life satisfaction (WVS ^h^), cortisol measurement, questions on worries, LOT-R ^i^, LCS ^j^	Significant reduction in depression and increase in happiness, life satisfaction, and psychological well-being index in the intervention group.
Haushofer & Shapiro [52]	GiveDirectly UBI program	Kenya	Both: periodic and lump-sum payments	✓	X	✓	RCT	*N* = 471 households	*N* = 469 households (‘spillover’ control); *N* = 432 households (pure control)	CES-D-20, PSS, questions on happiness and life satisfaction (WVS), cortisol measurement, questions on worries, LOT-R, LCS, Women’s Empowerment Index (measurement tool not specified)	Significant reduction in depression, worry, and stress and increase in happiness, life satisfaction, optimism, and psychological well-being index in the intervention group.
Haushofer & Shapiro [53]	GiveDirectly UBI program	Kenya	Both: periodic and lump-sum payments	✓	X	✓	RCT	*N* = 471 households	*N* = 469 households (‘spillover’ control); *N* = 432 households (pure control)	CES-D-20, PSS, questions on happiness and life satisfaction (WVS), cortisol measurement, questions on worries, LOT-R, LCS	Significant reduction in depression and stress and increase in happiness, life satisfaction, and psychological well-being index in the intervention group within 15 to 19 months after the start of the intervention.
Banerjee et al. [54]	GiveDirectly UBI program	Kenya	Both: periodic and lump-sum payments	✓	X	✓	RCT	*N* ≈ 22,600; 195 villages, aged over 18	*N* ≈ 11,000; 100 villages, aged over 18	CES-D-20, Locus of Control Scale (measurement tool not specified)	Significant reduction in depression among household heads in short-term and long-term intervention groups but not in the one-time payment group. No significant differences in the locus of the control.
Daidone et al. [55]	Livelihood empowerment against poverty program	Ghana	✓	X	X	✓	Qualitative and quantitative study	*N* = 646 households	*N* = 843 households (629 first comparison group, 214 additional to generate more statistical power)	Happiness scale (measurement tool not specified)	The likelihood of happiness increased by 16 percentage points in the intervention group compared to the control group (mainly due to smaller households and female-headed households). An important program effect on self-esteem, hope, and overall happiness reported in the qualitative part.

^a^ Center for Epidemiologic Studies Depression Scale, shortened version with 10 items [56]. ^b^ General Health Questionnaire. ^c^ Mental Health Inventory. ^d^ Geriatric Depression Scale. ^e^ Spillover control refers to control households within villages where the intervention was implemented. This was used to control for the so-called spillover effect, which describes the influence that the UBI and UCT transaction might have had on the well-being, behavior, and outcomes of households that did not directly receive this money [57]. ^f^ Center for Epidemiologic Studies Depression Scale, original version with 20 items [58]. ^g^ Perceived Stress Scale. ^h^ World Values Survey. ^i^ Revised form of the Life Orientation Test [59]. ^j^ Locus of Control Scale [60].

**Table 2:** Evaluation of the quality of individual included studies according to CASP.

Authors	Validity	Methodological Adequacy	Reporting of Results	Applicability	Research Quality *	Methodological Issues
American Research Institute [43] ^a^	3/3	2/3	2/3	2/2	9/11	Self-reporting used (bias). Due to the short duration of the program (24 months), the results may not have shown a measurable impact. Geographical remoteness may have contributed to poorer access to health and education services, potentially limiting the program’s effectiveness. The effects could be greater compared to richer areas due to the presence of very high levels of poverty. Expectations about the future were measured using only three self-invented questions, which are not part of a recognized questionnaire, making the results non-comparable with other studies.
American Research Institute [44] ^a^	3/3	2/3	2/3	2/2	9/11	The same shortcomings as in AIR (2014), as it is the same study, only reporting the effects of the program after 32 months.
Natali et al. [45] ^a^	3/3	2/3	2/3	1/2	8/11	Self-reporting used (bias). Given the diverse operationalization of happiness and subjective well-being, it would make sense for the study to include more standardized measures that assess these constructs. Missing baseline data at the start of the program prevent commenting on changes and the program’s impact on mental health indicators (measurements were only performed during the last waves, at 36 and 48 months). Lack of triangulation of the measured construct of happiness.
Baird et al. [46] ^a^	3/3	2/3	3/3	1/2	9/11	Self-reporting used (bias). Missing baseline data at the start of the program prevent commenting on changes and the program’s impact on mental health indicators (two rounds of questionnaire implementation after the program started, one used in both rounds, one only once). Inconsistent reporting and interpretation of results from tables to text. Non-comparability of groups due to different numbers of participants in each. Insufficient citation of sources for the mental health questionnaires used.
Kilburn et al. [47] ^a^	3/3	2/3	2/3	1/2	8/11	Self-reporting used (bias). Short time frame of the study. Changes in an individual’s criteria for a satisfying life are not necessarily related to the program.
Salinas-Rodriguez et al. [48]	2/3	2/3	2/3	2/2	8/11	Self-reporting used (bias). Missing baseline data at the start of the program prevent commenting on changes and the program’s impact on mental health indicators (the qualitative part of the study did not include pre-program measurements). Results are not generalizable—data were only obtained for older adults aged 70 to 74 living in rural areas, and we do not know the program’s effects on those older than 74 and those living in cities. Only short-term effects were studied (after 12 months), not long-term. Insufficient description of the qualitative part of the study—it is only stated that semi-structured interviews were thematically divided into four sections, but not all the questions used are listed.
	2/2 ^b^	3/4 ^b^	3/3 ^b^	0/1 ^b^	8/10 ^b^	
Kilburn et al. [49] ^a^	3/3	2/3	2/3	1/2	8/11	Self-reporting used (bias). Missing baseline data on adolescents/participants. Inconsistent reporting and interpretation of results from tables to text and inclusion criteria. Non-comparability of groups, as the experimental group included more orphans than the control group. Results are not generalizable, as the program is aimed at households with orphans.
Handa et al. [50] ^a^	3/3	2/3	2/3	1/2	8/11	Self-reporting used (bias). Non-comparability of groups, as the experimental group included more households than the control group. Factors other than parental SWB may contribute to changes in SWB in young people.
Haushofer and Shapiro [51] ^a^	1/3	1/3	2/3	0/2	4/11	Self-reporting used (bias). Participants were not completely blinded to group allocation. For measuring worries, they designed their own questionnaire, which lacked transparency. The sample was selected based on thatch roof data (the main and only criterion for determining low socioeconomic status). The final survey was conducted before some households received all transfers. The control group was included in the study only at the end of the program.
Haushofer and Shapiro [52] ^a^	3/3	0/3	3/3	2/2	8/11	Self-reporting used (bias). The sample was selected based on thatch roof data (the main and only criterion for determining low socioeconomic status). The effects of cash transfers on psychological well-being may be biased due to the spillover effect. The problem with analyzing the spillover effect is the later application of the criterion (one year later) for the control group. As a result, certain households were excluded from the final analysis at the end of the study. For measuring worries, they designed their own questionnaire, which lacked transparency. The control group was included in the study only at the end of the program.
Haushofer and Shapiro [53] ^a^	2/3	0/3	2/3	1/2	5/11	Self-reporting used (bias). The sample was selected based on thatch roof data (the main and only criterion for determining low socio-economic status). Participants were not completely blinded to group allocation. For measuring worries, they designed their own questionnaire, which lacked transparency. The method for measuring locus of control and calculating the psychological well-being and Women’s Empowerment Index is also unknown, limiting the validity of the results. The control group was included in the study only at the end of the program. The final survey was conducted before the program ended.
Banerjee et al. [54] ^a^	3/3	2/3	2/3	1/2	8/11	Self-reporting used (bias). Analysis of intervention during the COVID-19 pandemic, which may limit generalizability. Inaccurate or insufficient citation of the measurement instruments used limits the validity of the results.
Daidone et al. [55]	3/3	2/3	2/3	1/2	8/11	Self-reporting used (bias). Given the diverse operationalization of happiness, it would make sense for the study to include a standardized measure to assess this construct. Regional effects were not controlled—control households lived in different regions compared to program participants. Irregular payments and low values of the transfers could affect results.
	1/2 ^b^	3/4 ^b^	2/3 ^b^	1/1 ^b^	7/10 ^b^	

^a^ CASP version for randomized controlled trials. ^b^ CASP version for qualitative studies. * Consensus quality rating by two evaluators.

## Data Availability

No new data were created.
